# Digestive Organs

**Published:** 1906-02-24

**Authors:** 


### DIGESTIVE ORGANS.

(*Continued from page* 339.)

**Examination of the Fæces.**—In diseases of the liver and pancreas the condition of the fæces may give a useful aid to diagnosis, but the methods of accurate examination are too intricate for general adoption, and require not only a skilled observer, but a properly equipped laboratory. Cammidge,[5] whose work on the urine is now well known, has summarised his experience of 53 cases of biliary obstruction and pancreatic disease, and gives an account of his methods. In each case the reaction to litmus, the stercobilin, the total percentage of nitrogen, and the fat was estimated, and the presence of muscle fibre sought for microscopically. Briefly, his results show that in obstruction of the common bile duct high up in its course the reaction of the fæces is nearly always alkaline, while the amount of fat is not much above normal, the fatty acids being in excess of the neutral fats. When, however, the obstruction is low down, and the pancreatic secretion also interfered with, the results are opposite. The stools are then nearly always acid, and often there is considerable excess of fat, especially in cancer of the head of the pancreas. In these cases the neutral fats are usually in excess. Neither the search for muscle fibre nor the estimation of the nitrogen percentage gives very useful results, though undigested meat fibre and an excess of nitrogen are often found in pancreatic disease. Even when the stools were white in colour, stercobilin could always be detected in cases of chronic pancreatitis and obstruction; whereas in cancer of the pancreas it was completely absent in ten cases, and only present in the smallest traces in three. Cammidge's results suggest that useful information may sometimes be obtained by these somewhat elaborate methods, and, apart from this, the reaction and microscopic appearances of the stools should be more frequently determined. Fat globules, crystals, muscle fibre, &c., may be detected fairly easily, and a rough estimate of their excess or otherwise be formed.

**Causation of Jaundice.**—Much discussion has from time to time taken place as to the origin of the pigment deposited in the tissues in jaundice and its mode of entry into the blood. With the modern view that all jaundice is essentially obstructive Gerhardt[6] agrees, either by virtue of increased viscosity of the bile, or mechanical obstruction of the large ducts. He does not admit the existence of urobilin-jaundice, but thinks that bile pigments formed in the liver alone are responsible for the pigmentation. He allows, however, that blood destruction may occur in the tissues, only the disintegration products are all converted in the liver into bile pigment. In icterus neonatorum alone he considers that absorption of bile pigments from the meconium may occur in the intestine (urobilia). In a few rare instances he thinks that faulty processes in the liver cells may possibly cause the bile to pass into the blood-vessels instead of the bile capillaries, but of this he sees at present no actual proof. Finally he calls attention to the importance of cholangitis in the production both of gall-stones and simple "catarrhal" jaundice, quoting the experiments of Lüdke, who found that the blood serum in such cases not infrequently agglutinates the typhoid or colon bacilli.

**Treatment of Cirrhosis.**—A somewhat elaborate plan of treatment for cases of alcoholic origin has been formulated by Huchard and Fiessinger.[7] They begin with a month on milk diet, half a pint every two hours. Vegetable soup is then added and some cereals, and later still purées of lentils, peas, beans, etc., with baked potatoes and butter. Calomel in small doses (gr. $\frac{1}{3}$—$\frac{1}{2}$ in a week) is alternated every week with sulphate of soda—a teaspoonful every morning in a glass of water. At first, hot fomentations are applied to the liver every morning, with cold compresses during the day, and massage of the abdomen for ten minutes. An ointment containing various oleoresins and juniper may be rubbed in. As a biliary stimulant Robin prescribes benzoate and phosphate of soda with jaborandi, and to promote diuresis in ascites theobromine and sodium phosphate. The authors hold purging more efficacious than diuresis; they do not advise Talma's operation. If, as shown by daily weighing, the ascites is observed to increase, they recommend tapping. For epistaxis they advise mopping out the nostril with sterilised gelatine mixed with salt solution, and for bleeding from the alimentary tract they give calcium chloride and opium. Piles may be treated with tar ointment introduced into the anus or by warm wet compresses and tampons.

[5] Brit. Med. Jour., Oct. 28, 1905, p. 1102. [6] Med. Chron., Oct., 1905, p. 30. [7] Pract., 1905, ii., p. 423.

*(To be continued.)*

